# Brain–Computer Interface-Controlled Exoskeletons in Clinical Neurorehabilitation: Ready or Not?

**DOI:** 10.1177/15459683221138751

**Published:** 2022-11-25

**Authors:** Annalisa Colucci, Mareike Vermehren, Alessia Cavallo, Cornelius Angerhöfer, Niels Peekhaus, Loredana Zollo, Won-Seok Kim, Nam-Jong Paik, Surjo R. Soekadar

**Affiliations:** 1 Clinical Neurotechnology Laboratory, Neurowissenschaftliches Forschungszentrum (NWFZ), Department of Psychiatry and Neurosciences, Charité Campus Mitte (CCM), Charité – Universitätsmedizin Berlin, Charitéplatz 1, Berlin, Germany; 2 Unit of Advanced Robotics and Human-Centred Technologies (CREO Lab), University Campus Bio-Medico of Rome, Roma RM, Italy; 3 Department of Rehabilitation Medicine, Seoul National University College of Medicine, Seoul National University Bundang Hospital, Bundang-gu, Seongnam-si, Gyeonggi-do, Republic of Korea

**Keywords:** brain–computer interface (BCI), stroke, exoskeletons, motor recovery, clinical translation

## Abstract

The development of brain–computer interface-controlled exoskeletons promises new treatment strategies for neurorehabilitation after stroke or spinal cord injury. By converting brain/neural activity into control signals of wearable actuators, brain/neural exoskeletons (B/NEs) enable the execution of movements despite impaired motor function. Beyond the use as assistive devices, it was shown that—upon repeated use over several weeks—B/NEs can trigger motor recovery, even in chronic paralysis. Recent development of lightweight robotic actuators, comfortable and portable real-world brain recordings, as well as reliable brain/neural control strategies have paved the way for B/NEs to enter clinical care. Although B/NEs are now technically ready for broader clinical use, their promotion will critically depend on early adopters, for example, research-oriented physiotherapists or clinicians who are open for innovation. Data collected by early adopters will further elucidate the underlying mechanisms of B/NE-triggered motor recovery and play a key role in increasing efficacy of personalized treatment strategies. Moreover, early adopters will provide indispensable feedback to the manufacturers necessary to further improve robustness, applicability, and adoption of B/NEs into existing therapy plans.

## Introduction

Driven by advancements in sensor technology, wearable robotics, availability of computational capacities and other innovations such as 3D-printing, brain–computer interface (BCI)-controlled exoskeletons are rapidly evolving as powerful tool to restore or improve autonomy and quality of life across various disorders of the nervous and motor system. BCIs were already conceptualized in the early 1970s.^1^ By translating brain activity into control signals of external devices they enable volitional and intuitive control of computers or machines,^2^ for example, exoskeletons or prostheses. Moreover, BCI technology can be also used to drive electric stimulators, for example, activating peripheral muscles in the form of functional electric stimulation (FES).^3^ It was shown that individuals suffering from severe muscle paralysis can use BCI technology across various scenarios, for example, to control a robotic arm,^4,5^ a motor-driven hand orthosis,^6^ or a neuromuscular FES device to reach, grasp, and manipulate different objects of daily living.^7,8^

Biosignals used for BCI applications are either measured invasively by hardware components surgically inserted directly into the brain,^4,5^ or non-invasively from the surface of the scalp.^6,9^ Implantable BCIs typically record local field potentials or single and multi-unit neuronal cell activity allowing for versatile and high-dimensional control of robotic arms and fingers,^5^ or for direct electric stimulation of peripheral nerves.^10^ A less invasive approach uses electrocorticography, but still requires craniotomy.^11^ Despite efforts to reduce tissue damage, surgical risks have to be carefully balanced with the anticipated individual benefit of implantation. Besides the risk of infections and bleedings, any repair or removal of implantable devices requires additional surgery. Also, in intracortical chronic implantations foreign body response may result in glial scar formation and neuronal loss^12^ that can impact stability and reliability of BCI decoding over time. Moreover, operant control of multi-dimensional robotic actuators, for example, a full-body exoskeleton, requires extensive training, and there are only very few such actuators available, most of them not certified or approved by the U.S. Food and Drug Administration (FDA) or cleared in compliance with the European Union’s Medical Device Regulation (MDR). Although very powerful, it is thus likely that in near future implantation of BCIs will be reserved for individual cases or research purposes.

In contrast, non-invasive BCIs, for example, based on electroencephalography (EEG) or near-infrared spectroscopy,^13^ allow for brain activity recordings in a risk-free and more accessible manner. This makes particularly EEG-based BCIs most promising for broader clinical application, despite their limited capacity to decode, for example, different grasp types, from electrical brain signals. Novel sensors, for example, optically-pumped magnetometers^14^ or nitrogen-vacancy magnetometers, might overcome this limitation, but are at a very early stage and require elaborate magnetic shielding.

Due to its broad availability, EEG became the most commonly applied recording technique in BCI applications for motor rehabilitation so far.^15^ Besides evaluating evoked brain responses, for example, steady state visually evoked potentials for action selection,^16^ voluntary modulations of sensorimotor or mu-rhythms are the best established features for BCI control in paralysis. It was shown that even stroke survivors with severe chronic paralysis can successfully learn to operate such motor BCIs.^17^ Here, operant control of cortical activity is typically quantified as event-related-desynchronization or synchronization (ERD/ERS) and translated into online control of external devices. While early clinical BCIs were mainly conceptualized as assistive tools, for example, to restore communication or movements (*assistive* BCIs), it was shown that systematic and repeated use of BCIs can result in functional and structural plasticity of the nervous system associated with restoration of motor function, for example, after stroke^18^ or spinal cord injury (SCI)^19^ (*restorative* BCIs). Such restorative BCI applications constitute nowadays an important pillar of clinical BCI research.^15^

Given that first assistive motor BCIs have been introduced more than 2 decades ago and first evidence for their clinical benefit was presented more than 10 years ago, the question arises why BCIs have not yet arrived in clinical routine care of severe paralysis following stroke or SCI.

There are 2 main reasons: (1) Accuracy and reliability of non-invasive BCI systems, typically ranging between 65% and 80%, were too low for assistive use, (2) The effect size (eg, standardized mean difference, SMD) of BCI training applied over several weeks on motor recovery was not sufficiently high and inconsistent across studies (SMD of 0.16-1.20),^15,20-22^ and interindividual motor recovery was too variable (eg, mean difference in the Fugl–Meyer-Assessment for upper extremity (FMA-UE) score pre–post intervention of 6.3 to 13.2 points across subacute to chronic stroke survivors)^23^ to justify costs and efforts of daily BCI training. In this context, it is important to note that available diagnostic instruments to assess functional improvements after BCI training are optimized for sensitivity in moderate to mild paralysis (eg, FMA) and cannot adequately assess the effectiveness and effect size in patient groups with minimal residual hand function.^24^ Moreover, daily BCI training can be very tiring and requires high levels of motivation, especially if the system’s feedback is not sufficiently rewarding over time.

To tackle these challenges, over the last years, we have introduced and validated some novel brain/neural control approaches,^6,25^ and conceptualized a BCI neurorehabilitation framework that combines both the assistive and restorative dimension of BCIs^26^ to sustain high levels of motivation and maximize the rehabilitative impact. At the core of this development lies the implementation of non-invasive *hybrid* systems merging brain and neural signals, for example, related to eye movements, to increase accuracy and reliability during assistance.^6^ This enables individuals with severe finger paralysis to operate such brain/neural assistive devices in real-life situations, for example, to eat and drink in an outside restaurant.^6^ Here, the additional use of neural signals related to horizontal oculoversions (HOV) substantially increased safety during operation, even in very noisy and uncontrolled environments. Moreover, the immediate increase in autonomy and independence motivated the users to continue operating such brain-controlled system. Another decisive prerequisite lies in the availability of lightweight and portable exoskeletons that can deal with spasticity and enable the users to engage in activities of daily living (ADLs), such as cooking, dressing, or engaging in personal hygiene. Carrying out such ADLs is often impossible for stroke or SCI survivors without assistance. Despite some existing treatment strategies for SCI survivors, for example, tendon transfer surgery that can restore motor function to a certain extent, pre-lesion hand function cannot be achieved.^27^ In stroke, approximately 30% to 40% of stroke survivors do not show sufficient motor function to engage in established rehabilitation strategies, for example, constraint-induced movement therapy (CIMT) or standard occupational practice.^28^ For these patients, there is currently no standardized treatment option available to restore bimanual ADLs. We, thus, reasoned that repeated use of a brain/neural exoskeleton (B/NE) restoring bimanual ADLs during physiotherapy would fill a critical gap in the rehabilitation pipeline for these severely affected patient populations.

## The Promise of BCI-Enabled Neurorehabilitation

Early conceptions of BCIs focused on the possible assistive value of such technology, translating thoughts into action. Consequently, first prototypes had labels such as “thought translation device”^29^ and were successfully applied as a spelling device in patients with locked-in syndrome.^30^ After Fetz^31^ demonstrated that single motor neurons can be operantly conditioned to increase their firing rates, the same principle was used to develop implantable BCIs to control robotic arms or prostheses.^32^ Successful demonstrations of such technology have not only inspired science fiction novelists, but also raised the hope that BCIs would eliminate the burden of severe paralysis 1 day. But where are we today in this matter? Have BCIs finally come of age to deliver on its promises?

While implantable BCIs have proven an impressive range of versatility, particularly when combined with advanced machine learning algorithms and intelligent robotics, non-invasive BCIs remain rather limited due to low information content of brain signals recorded from the surface of the skull and their susceptibility to artifacts. Innovative sensors may overcome this limitation 1 day, but their implementation into clinical BCI applications will still require many years. Nonetheless, non-invasive BCIs may deliver on their promise from a different, not much anticipated, angle.

With the advent of modern neuroimaging, notions of the brain’s remarkable capacity to reorganize and recover, even from severe damage, increasingly substantiated. The ability for operant learning critically depends, however, on the availability of feedback signals indicating level of success. In case of an acute brain or spinal cord lesion, inflammation and swelling can lead to complete loss of motor function. In such situation, no feedback signals indicating level of success are generated, that is, intended movements do not have any consequences. Even worse, lack of any movement may additionally weaken the remaining synaptic connections within the existing sensorimotor loop, in many cases chronically. *Restorative* BCIs aim at reversing this process by providing feedback to activate neural assemblies in an associative manner^26,33^ similar to paired-associative stimulation.^34^ After early indications that voluntary modulation of the sensorimotor rhythm (SMR) can improve stroke outcome, larger randomized controlled trials corroborated this finding.^15,20-22^ Despite these large clinical trials, the exact mechanisms underlying BCI-triggered motor recovery are not yet fully understood. Neurophysiological and neuroimaging studies showed structural and functional normalization of the damaged hemisphere (eg, increase of intrahemispheric connectivity, stronger ERDs of alpha and beta bands during imagined movements of the paralyzed hand) when contingent feedback of ipsilesional brain activity was provided, but not when random feedback was provided.^18,35^ It was also suggested that pre-trial motor-related cortical potentials or functional connectivity measures in sensorimotor areas may be a neural correlate of motor recovery in stroke.^36^ The correlation between such neurophysiological measures and functional clinical outcomes (eg, the FMA-UE score) corroborated the clinical relevance of BCI-based interventions.^8,37^ It is noteworthy that efficacy of BCIs depended on the type of sensory feedback: somatosensory and proprioceptive feedback, for example, administered via a robotic orthosis, was found to be superior in facilitating neural reorganization in the brain when compared to visual presentation of hand movements only.^38^ Since stroke survivors with severe cortical and subcortical damage may not present with consistent ipsilesional ERD/ERS modulations, also BCI training using the contralesional, unaffected hemisphere was investigated and showed promising results.^39^ When comparing different BCI feedback devices (visual feedback device, robot and arm orthosis moving the entire arm or opening and closing the paralyzed hand, or FES), FES proved superior to other approaches.^21,22^ Provided that FES can restore ADLs to the level of versatile exoskeletons, the previously mentioned motivational aspect related to restoration of bimanual ADLs makes repeated use of B/NEs or brain-controlled FES the most promising strategies for BCI-enabled neurorehabilitation.^40^

By allowing paralyzed patients to securely grasp and manipulate different objects of daily life and engage in ADLs, B/NE or brain-controlled FES can also increase the involvement of the whole affected limb and body in training sessions. Besides unspecific benefits of mobilization, also so-called *learned non-use* can be avoided or counteracted, even in stroke survivors with no residual movements. Learned non-use is a common phenomenon in stroke survivors who over time prefer the use of the unaffected side due to initial failure to use the affected side.^41^ Since it was found that learned non-use is not only correlated with low autonomy, but also linked to lower levels of quality of life and increased levels of anxiety and depression,^42^ counteracting learned non-use with BCI-enabled technology restoring bimanual ADLs early in the rehabilitation phase may reduce post-stroke depression currently affecting every third stroke survivor.^43^

In the following, we focus on B/NEs, describe the state-of-the-art and elaborate on the current challenges to make these systems widely accessible.

## B/NEs for Clinical Application: Where Are We?

**Figure 1.**
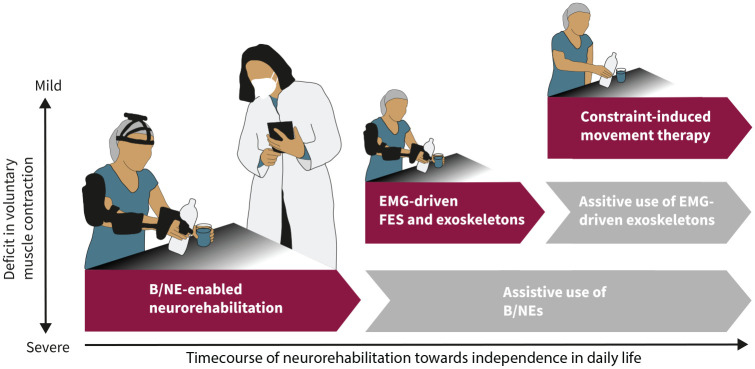
Neurorehabilitation pipeline that integrates brain/neural exoskeletons (B/NE) to allow for bimanual activities of daily living (ADLs) in severe paralysis. If B/NE training results in restoration of decodable electromyographic (EMG) activity in the affected limb, EMG-driven functional electric stimulation (FES), and/or exoskeletons are used to augment the rehabilitation effect. If not, use of assistive B/NEs or otherwise controlled exoskeletons is continued. If EMG-driven FES and/or exoskeleton training results in restoration of functional movements, constraint-induced movement therapy is applied to augment the rehabilitation effect. If not, use of EMG-driven assistive exoskeletons is continued.

Over the last years, important technological improvements have been made that pave the way for the implementation of B/NEs in neurorehabilitation. Building on these improvements, we envision a neurorehabilitation pipeline that uses brain-controlled technology early in the rehabilitation process (Figure 1) followed by other approaches, such as electromyographic (EMG)-controlled FES, as soon as decodable EMG activity can be detected, and CIMT, as soon as functionally relevant finger and hand movements are present.^44^

Implementation of such BCI-enabled neurorehabilitation pipeline will critically depend on early adopters, that is, research-oriented physiotherapists or clinicians who are open for innovations. In the following, we will depict the state-of-the-art in mobile EEG, wearable exoskeletons, and brain/neural control algorithms to illustrate B/NEs’ technology readiness level.

### Reliable B/NE Control

Successful implementation of B/NEs requires accurate and reliable classification of brain/neural signals. In other words, the intention of users to move or not to move needs to be accurately decoded from their brain/neural activity and converted into a control command for the exoskeleton. Due to the EEG’s low signal-to-noise ratio and non-stationarity, as well as the limited information that is decodable from non-invasive brain recordings, state-of-the-art linear classifiers for EEG-BCI systems achieve a classification accuracy of 60% to 80% only.^45^ Although this classification accuracy ranges substantially below levels that are desirable for assistance in daily life (usually close to 100%), this low rate is sufficient to trigger operant learning.

Besides linear classifiers also other algorithms, for example, convolutional neural networks (CNN), were introduced for BCI classification.^46^ Hybrid architectures, usually combining a CNN with a different deep learning algorithm, have outperformed state-of-the-art methods.^47,48^ However, applying neural networks for BCI classification requires large amounts of training data.^48^ While such approaches may reduce calibration time, it is unlikely that their accuracy and reliability will be sufficient to drive assistive exoskeletons with brain signals only. Moreover, adaptive algorithms that optimize for control performance were shown to reduce BCI learning.^49^ Given that BCI learning (eg, quantified as increasing ability to modulate ipsilesional ERD/ERS) reflects functional and structural plasticity, improving the speed and effectiveness of BCI learning (not performance) was proposed as priority for the field, for example, using multimodal or multi-stage approaches.^50^

In everyday life environments, in which users are freely moving, muscle artifacts pose another critical challenge. To establish reliable BCI control under such conditions, several software and hardware solutions have been introduced over the last decade to de-noise EEG data. In particular, the development of adaptive filters, for example, adapting to the dynamic characteristics of muscle artifacts, further advanced the ability to remove signal artifacts in real time.^51^

To allow for BCI-enabled assistive applications despite insufficient BCI decoding performance (<80%), a combination of biosignals, for example, EEG and electrooculography (EOG), was proposed.^52^ Here, in the context of brain/neural control of hand exoskeletons, hand opening/releasing movements and interruption of unintended closing movements could be stopped by HOV, which significantly increased the system’s reliability and safety. By decoding HOV from scalp electrodes, biosignal recording sites could be minimized without reducing the system’s reliability (resulting in EOG detection at 100% sensitivity in 16 out of 18 study participants and overall control performance of 87.91% ± 31.88%),^9^ rendering B/NEs practical and sufficiently safe for assistive applications. For BCI-controlled lower limb exoskeletons shared control approaches that include automatic context-dependent decision-making were suggested.^53^

### Wireless and Portable EEG Systems

For many decades, EEG was mainly used as a diagnostic tool in neurology and psychiatry, for example, to detect abnormal brain activity or to characterize epileptic seizures. For such applications, EEG systems were usually stationary and optimized for high signal quality using conductive gels and gold or silver electrodes. Driven by miniaturization and advances in wireless technology, portable EEG-amplifiers were developed that allow now for mobile recordings outside the laboratory. For example, 32-channel recordings are now possible with EEG amplifiers that can be attached to the user’s head and carried around. To reduce preparation time, dry electrodes that typically use metallic spike arrays^54^ and solid gel^55^ electrodes were introduced that do not require gels or fluids. However, these electrodes require direct contact with the scalp and often come with lower signal quality.^56^ Another alternative to the conventional wet electrodes uses saline soaked sponges (eg, *R-net* by Brain Products, *waveguard net* by ANT Neuro, or *GT Cap Gelfree-S3* by Greentek) that also reduce preparation time and require no hair washing after application.

Another important advancement to increase robustness of B/NE applications was the reduction of necessary electrodes. By implementing a novel EEG/EOG control strategy that uses only scalp electrodes placed near cortical sensorimotor areas, for example, integrated into a headset, applicability and practicality of non-invasive brain/neural recordings could be substantially improved.^9^ Taken together, these advances make fast and reliable EEG recordings for assistive BCI applications possible.

### Lightweight, Portable, and Versatile Exoskeletons

In analogy to EEG systems, robotic exoskeletons are increasingly becoming lightweight and wearable. In the past decades, robotic exoskeletons were mostly stationary, enabling limited movement, that is, finger extension/flexion, but no functional grasping.^57^ First systems allowing manipulation of objects outside the laboratory still came with bulky control boxes and battery units that did not allow patients to move around freely.^6^ Building on various technological advancements, a new generation of portable and versatile exoskeletons has emerged. Depending on the characteristics of construction, 3 distinct types of these exoskeletons can be distinguished: rigid, soft, and hybrid exoskeletons.^57^ Exoskeletons with rigid components can typically exert high forces and can be controlled with great accuracy. However, such systems are often obtrusive and heavy (eg, MyoPro by Myomo, ~1.600 g), increasing the risk of injuries. In contrast, soft exoskeletons endowed with continuous bending capability, are less bulky and lighter (eg, Neomano by Neofect <65 g). They consist of flexible or elastic materials, and many are designed as a glove. This makes soft exoskeletons more adaptable to the individual body shape and more comfortable to use, but also reduce control accuracy and ease of mounting.

Designing exoskeletons for stroke survivors with severe paralysis is particularly challenging due to spasticity and muscle atrophy. To restore motor function, exoskeletons must be adapted to the individual anatomy, anthropometry as well as movement range and required forces. For example, exoskeleton joints must be aligned with the finger joints to prevent injuries, and exoskeletons must be adjustable to fit different hand sizes. Depending on the level of spasticity, different forces may be required to move the hand and fingers despite antagonistic muscular tone. All these factors show great interindividual variability and may change over the course of rehabilitation treatment.

Besides miniaturization of actuators and electronics, availability of 3D printing technology has strongly promoted the development of user-tailored exoskeletons (eg, *exomotion*© from HKK Bionics) and reduction of related production time and costs.^58^ Nevertheless, all systems must fulfill the requirements for basic safety and performance of the FDA or MDR to receive medical device certification. Potential risks, such as device malfunction resulting in unintended movement, soft tissue damage, joint misalignment, electrical or mechanical hazards, or user error need to be mitigated and carefully considered. Of the numerous examples of exoskeletons developed for medical purposes, only few are commercially available, for example, the *HandyRehab* device by Zunosaki Ltd., the *Gloreha Sinfonia* glove by Idrogenet srl., the *Neomano* device by Neofect, the *mano* by Emovo srl., the *Armeo Power/Spring/Manovo Spring* by Hocoma AG, or the *MyoPro*^®^ device by Myomo Inc. for the upper extremity, and the *Ekso, HAL, Indego, REX, ReWalk, ALEX, Atalante*, and stride management assist (*SMA*) system^59^ for overground walking beyond assistance on a treadmill. However, none of these systems has been marketed with the option for brain/neural control, so far.

## Translation of B/NEs Into Routine Clinical Care: What is Missing?

In April 2021, the FDA authorized marketing of the first brain-controlled hand exoskeleton for neurorehabilitation (*IpsiHand*, Neurolutions). This device is the first medical product envisioned for BCI-enabled hand rehabilitation of stroke survivors and represents the first tangible attempt to move B/NEs into rehabilitative care. The product comprises both the hardware and software that is necessary for BCI-enabled hand motor training. Clinical data collected from 40 patients in a 12-week open-label, uncontrolled clinical trial suggested that the system is safe and effective (unpublished data submitted to FDA). Adverse events included minor fatigue and discomfort as well as temporary skin redness. The system mobilizes the index and middle finger upon desynchronization of mu-rhythms recorded by a dry electrode headset. The device has not been approved for patients with severe spasticity or rigid contractures in the wrist or fingers, and cannot be used to perform ADLs, for example, grasping a bottle or holding a toothbrush. Even if this system does not yet restore (bimanual) ADLs, which would be important as outlined in section 2, it demonstrates that the technology readiness level of brain-controlled exoskeletons is now sufficient to incorporate them into clinical care. Wide adoption of B/NE-based treatment, however, will critically depend on early adopters who are open for innovation. Accessibility as well as integration into existing treatment concepts and therapy plans will be associated with several challenges outlined in the following sections.

### Patient Stratification and Personalized Treatment

Due to the heterogeneity of stroke and SCI survivors regarding age, type and severity of stroke, lesion location, time since injury, biological phenotypes and integrity of corticospinal tract and subcortical brain structures, rehabilitation plans often require personalization to the individual motor and cognitive functions.^23^ For instance, B/NE training duration and intensity should be tailored to the patient’s individual capacity to maximize the training effect while minimizing the risk of adverse effects. Here, monitoring physiological biomarkers predicting mental exhaustion, for example, heart-rate variability, galvanic skin response, or respiration rate could improve training schedules.^60^ Moreover, introducing visual neurofeedback training of ipsilesional mu- or SMR-ERD in the earliest phase of rehabilitation could strengthen motor representations that are weak and fluctuant as a direct result of stroke or have been negatively affected by learned-non-use.^61^ Currently, it is unclear which patients benefit the most from BCI-enabled neurorehabilitation training and which training parameters are optimal.^23^ Presence of motor-evoked potentials in the affected limb, functional connectivity, as well as synchronicity between sensorimotor regions were found to be promising predictors,^62^ but larger clinical trials are needed to confirm these findings.^63^ Clear definition of inclusion and exclusion criteria is needed to promote the adoption and the benefit of B/NE treatment, and to determine the Minimal Clinically Important Difference. Heterogeneity of patient populations renders large clinical trials investigating specific mechanisms of recovery and predictors of rehabilitation very challenging, however. Thus, only large hypothesis-generating multicenter studies might provide the necessary data sources so that machine learning and artificial intelligence-enabled research methods can contribute to elucidate such mechanisms.

### Integration Into Existing Treatment Plans

Another key challenge to promote B/NEs into routine clinical care relates to their integration into existing treatment concepts and plans that vary across different rehabilitation schools and centers. Thus, general suggestions on how to integrate B/NEs into rehabilitation pipelines^61^ must be adapted to the specific clinical environments. Here, application of B/NEs critically depends on early adopters that are willing to take an extra effort to change existing workflows and to promote their use among clinicians and end-users. These early adopters, who usually account for less than 15% of a group,^64^ need institutional support and the required resources by their management. Well trained and supported early adopters will not only ensure safe and effective application of B/NEs, but also promote technology acceptance by the end-users and their relatives. From a technical point of view, preparation time of B/NEs must be minimal (in the minutes range) and their robustness high. This is challenging because BCI-enabled systems often require lengthy calibration procedures, and insufficient calibration reduces robustness. Moreover, user-friendliness is another important factor for the adoption of B/NEs. For instance, end-users should be able to operate the system without technical expertise. Here, close collaboration between early adopters, end-users, and the manufacturer will be crucial for continuous optimization of user-friendliness.

### Commercialization of B/NEs

Although BCI-enabled technology, such as B/NEs, would effectively address an important medical need of a very large and growing patient population, investors often shy back from the complexity of bringing such technology to the market. Besides the necessity of receiving certification or approval of the responsible authorities, which is a time-consuming and costly process, mechanisms or pathways for distribution of B/NEs are limited. Another critical factor is reimbursability. Since health insurances only reimburse the costs for medical devices or treatment upon demonstration of a clear, evidence-based benefit for the insured party, it may take many years before innovative treatments are finally included in the insurance’s catalog of covered services. However, since assistive exoskeletons are medical aids, some insurances were forced to cover the costs for such device by the social welfare court although the device was not listed in the medical aids register. This mechanism may motivate companies to engage in developing and commercializing assistive exoskeletons that could be also used for restorative BCIs.

## The Future of BCI-Enabled Neurorehabilitation: What’s Next?

Progress in BCI-enabled neurorehabilitation greatly capitalizes on the latest advancements and investments in neurotechnology. Since 2005, the number of neurotechnology patents has increased by more than 500%^65^ and BCI-developing companies such as Neuralink attract broad public attention. While implantable solutions will most likely remain reserved for a small fraction of stroke and SCI survivors in the coming years, non-invasive BCI technology is now ready for broad clinical translation. Currently, it is less the BCI component but more the lack of adaptable and robust exoskeletons that pose a bottleneck in implementation, particularly exoskeletons that restore ADLs. Providing the capability to perform ADLs is not only important to sustain a high level of motivation and to foster generalization of learned skills into daily life, but also to increase self-efficacy during therapy which was found to be correlated with rehabilitation outcome.^66^ Motivation and self-efficacy also affect treatment engagement into physiotherapy after completion of inpatient rehabilitative care.^67^ Moreover, the use of robotic exoskeletons might improve quality of life by reducing the severity of secondary health issues, for example, pain, spasticity, and autonomic dysfunction.^53^

**Figure 2.**
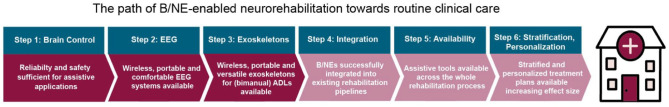
Translating brain/neural exoskeletons (B/NE) into routine clinical care. While the technical prerequisites have been established, including reliable brain/neural control paradigms, portable/wireless electroencephalography (EEG) and wearable exoskeletons, broader adoption will depend on successful integration into existing rehabilitation pipelines, availability across the whole rehabilitation process and further stratification and personalization of treatment plans maximizing effect sizes of clinical interventions. Abbreviation: ADLs, activities of daily living.

The main risks opposing successful adoption are (1) unmet user expectations, for example, because the system’s robustness or assistive function are insufficient, (2) lower effect size in motor rehabilitation than anticipated, (3) lack of availability across the whole rehabilitation process (particularly, when transitioning from inpatient to ambulatory treatment), and (4) delays in reimbursability of B/NE treatment. To mitigate the first risk, close interactions between early adopters, end-users, and the manufacturers will be crucial to improve user-friendliness and resolve unforeseen issues that limit the system’s continuous operational readiness. Integration into a sound rehabilitation pipeline will be important that also offers the perspective to continue the use of assistive tools if rehabilitation of voluntary movements fails. Once B/NEs reach sufficient robustness in clinical environments, another factor that will greatly accelerate their adoption will be the possibility for home use. This step should not be underestimated, however, because the risk of operating errors might potentiate. Embedding B/NEs in a comprehensive digital training environment could reduce this risk. Availability of data from large clinical samples will help to further elucidate the underlying mechanisms and predictors of B/NE-related motor recovery and allow for implementation of more stratified and personalized treatment plans (Figure 2).

Moreover, BCI learning could be accelerated by non-invasive brain stimulation (NIBS) techniques.^68^ Neuroimaging studies showed that BCI-guided robot-assisted upper-limb training in chronic stroke can lead to functional reorganization between ipsilesional motor regions (M1 and SMA) and contralesional areas (SMA, PMd, SPL). Moreover, such training also resulted in increased interhemispheric functional connectivity among the sensorimotor areas.^69^ These neural substrates could be targeted by NIBS such as transcranial direct current stimulation, transcranial magnetic stimulation, or adaptive brain state-dependent stimulation^70^ to maximize the efficacy of BCI-enabled neurorehabilitation. Recently, a real-time compatible stimulation artifact suppression algorithm was introduced that allows for millisecond-to-millisecond precise targeting of brain oscillations, for example, mu- or SMR, during BCI control.^71^ Such an approach that offers the possibility to selectively enhance or inhibit neural activity might help to uncover the underlying mechanisms of BCI-enabled motor recovery. Moreover, real-time adjustment of stimulation parameters depending on functional activation of the brain might also allow for maximizing the efficacy of combined NIBS and BCI-enabled neurorehabilitation.^70^

## Conclusions

BCI-enabled technology can facilitate motor recovery, thus providing a novel and powerful tool for motor rehabilitation after stroke or SCI. B/NEs that allow for performing ADLs may increase and sustain motivation, and consequently improve therapeutic efficacy. Despite technological readiness and first commercial availability of BCI-enabled rehabilitation systems, many challenges have yet to be mastered to ensure broad adoption of BCI-enabled technology into routine clinical care. Promotion of B/NEs and their implementation into existing treatment concepts critically depend on early adopters and institutions that are open for innovation.
